# Pomegranate disease detection and classification dataset for deep learning applications: A case study from Halabja city

**DOI:** 10.1016/j.dib.2025.112298

**Published:** 2025-11-20

**Authors:** Bashdar Abdalrahman Mohammed, Peshraw Ahmed Abdalla, Sirwan M Aziz, Hiwa Omer Hassan

**Affiliations:** a Department of Computer Science, College of Science, University of Halabja, Halabja, Kurdistan Region, Iraq; b Department of Computer Science, Darbandikhan Technical Institute, Sulaimani Polytechnic University, Darbandikhan, Kurdistan Region, Iraq; c Department of Computer Science, College of Science and Technology, University of Human Development, Sulaymaniyah, Kurdistan Region, Iraq

**Keywords:** Pomegranate, Fruit disease, Image dataset, Plant pathology, Computer vision, Disease classification, Halabja, Precision agriculture

## Abstract

Timely and accurate detection of pomegranate fruit diseases is critical for minimizing crop losses, preserving fruit quality, and supporting sustainable agricultural practices. This study introduces the Halabja Pomegranate Fruit Disease Image Dataset, a systematically compiled collection of images from orchards in one of Iraq’s major pomegranate-producing regions. The dataset comprises 2178 original images and 28,314 augmented images, categorized into four specific classes: ectomyelois ceratoniae, colletotrichum spp., sunburn, and healthy fruit samples. To create an ecological setting and ensure significant class variation, images were captured in natural outdoor environments. A standard preprocessing step was applied, which involved resizing all images to 512×512 pixels and using several image augmentation techniques to improve the flexibility and robustness of machine learning models. The unique characteristics of this dataset make it highly suitable for developing machine learning and deep learning models aimed at plant disease detection and other computer vision tasks in precision agriculture. Its contextual relevance and content diversity make it valuable for building an effective diagnostic tool capable of functioning in real field conditions.

Specification Table

**Table:** 

Subject	Artificial Intelligence and Computer Vision Applications in Precision Agriculture
Specific subject area	Pomegranate fruit disease classification using deep learning techniques in precision agriculture.
Type of data	Image (.JPG format) JPG processed, augmented (PNG) image files
Data collection	A total of 2178 images of pomegranate fruits were collected from orchards located in Halabja city. The images were captured using a Sony ILCE-7RM4 camera under natural lighting conditions to represent real-world field environments accurately. Fruit samples were selected based on observable disease symptoms, and a professional botanist validated their classification. The dataset is categorized into four distinct classes: Colletotrichum spp. (anthracnose), Ectomyelois ceratoniae (fruit borer), sunburn, and healthy fruit.
Data source location	Pomegranate orchards in Halabja city, Kurdistan region, Iraq (location code: 46,018).
Data accessibility	Repository name: Zenodo Data identification number: 10.5281/zenodo.15856012 Direct URL to data: https://zenodo.org/records/15856012 Halabja Pomegranate Fruit Disease Image Dataset. Zenodo [1].
Related research article	None

## 1 Value of the Data

• **Regional Uniqueness:** This dataset is the first publicly available collection of pomegranate fruit disease images from Halabja, in the Kurdistan Region of Iraq, an area renowned for its high-quality pomegranate production. Its distinctive climate and farming conditions make this dataset valuable for studying region-specific plant diseases.
• **Agricultural Application:** The dataset enables the early and precise detection of pomegranate diseases, thereby minimizing crop losses, maintaining fruit quality, and promoting sustainable farming practices. It provides real-world data that can benefit both farmers and agricultural researchers.
• **Scientific Resource:** Researchers in plant pathology, agriculture, and data science can use this dataset to study disease symptoms observed directly on pomegranate fruits, enabling accurate disease analysis and supporting experimental research.
• **Advancing Technology:** This dataset enables the development and testing of artificial intelligence models, computer vision tools, and automated systems for disease detection and classification in fruit crops.
• **Broader Reusability:** The dataset is useful for local and international researchers, agricultural organizations, and policymakers seeking to enhance disease management practices for pomegranate farming and related crops.

## 2 Background

Pomegranate (Punica granatum L.) is a long-lived fruit-bearing plant indigenous to the Middle East and various regions of Asia. It has been cultivated for centuries owing to its high nutritional content, therapeutic properties, and economic importance[2]. The fruit is particularly valued for its abundance of antioxidants, essential vitamins, and bioactive compounds, contributing to its strong demand in both domestic and global markets [3]. Halabja, a city in the Kurdistan region of Iraq, located in the northeastern part of the country, has gained recognition for producing some of the finest pomegranate varieties. The unique flavor, vibrant color, and overall quality of the fruit from this region are attributed to favorable climatic conditions, traditional cultivation practices, and fertile soil [4]. Halabja is a city renowned for high-quality pomegranate production, yielding over 30,000 tons annually across approximately 10,000 acres of cultivated gardens.

Although pomegranate holds substantial agronomic and economic importance, its cultivation is increasingly challenged by a range of biotic stress factors, most notably fruit diseases resulting from fungal pathogens, bacterial agents, and insect infestations [5]. These diseases result in significant yield losses, reduce fruit quality, and increase post-harvest rejection rates, thereby directly impacting the income of smallholder farmers and the regional agricultural economy. Early and accurate disease detection is therefore essential for effective crop management, timely intervention, and prevention of large-scale outbreaks.

Traditionally, farmers or Other agricultural specialists identified the diseases manually by relying on their vision. Although this is the standard way of doing things, it is subjective by nature, labor-intensive, and suffers from the disadvantage of inconsistencies, as experts and different environmental factors differ [6]. Due to the growing access to machine-learning and digital imaging approaches, there has been a paradigm shift in utilizing computerized systems to analyze plant diseases [7]. The systems are very dependent on the existence of high-quality, annotated datasets that cover a rich variety of symptoms across varied field conditions of the disease [8].

Most existing plant disease datasets focus on major crops, such as wheat, maize, and rice, with limited attention to specialty crops like pomegranate. This absence of localized, crop-specific data hinders the development of accurate diagnostic tools and regionally adapted disease management strategies. To address this gap, this study introduces a new dataset for pomegranate fruit diseases. It captures real-world symptoms and environmental variability, supporting AI-driven diagnosis and the advancement of precision agriculture in the region.

## 3 Data Description

This dataset contains 2178 high-resolution images of pomegranate fruits. The images were captured using a Sony ILCE-7RM4 camera with a resolution of 7952 × 4472 pixels, an sRGB color profile, a 31 mm focal length, and a horizontal resolution of 350 dpi (see Table 1 for details). These specifications ensure high visual quality and accurate symptom representation, making the dataset ideal for fine-grained computer vision tasks. The primary objective is to facilitate the detection and classification of common pomegranate diseases using machine learning and AI-based methods.

**Table 1:** Specifications of the camera device and image properties used in dataset acquisition.

Sl. No.	Particulars	Description
1	Camera Maker	SONY
2	Camera Model	ILCE-7RM3A
3	Dimensions	7952×4472
4	Resolution	350 dpi
5	Bit depth	24
6	F-stop	f/5.6
7	Exposure time	*1/160 s*
8	ISO speed	ISO-160
9	Exposure bias	0 step
10	Focal length	31 mm
11	Flash mode	No flash, Compulsory
12	Metering mode	Pattern

Each image is labeled according to its respective class to facilitate supervised learning tasks such as classification, object detection, and segmentation. The inclusion of both healthy and diseased samples allows for balanced model training and enhances the dataset’s applicability for both binary and multi-class classification problems.

Moreover, although a few images contain minor quality variations due to environmental or technical constraints (e.g., uneven lighting or slight blurring), these were deliberately retained to preserve the natural complexity of the field conditions. This approach enhances the dataset’s realism and improves its utility for developing models intended for deployment in real agricultural environments.

**Fig. 1:**
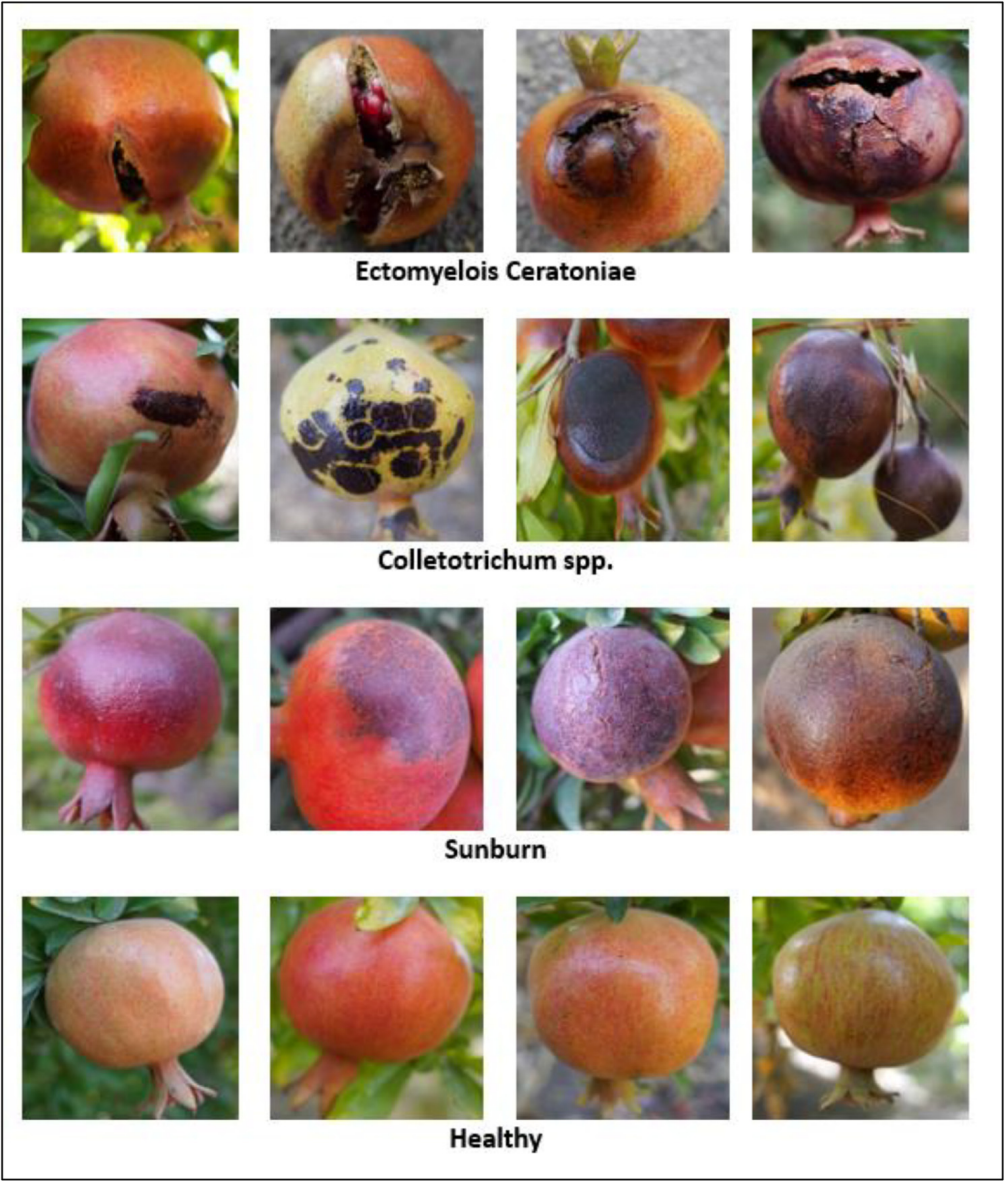
Representative images of pomegranate fruit disease classes.

The dataset is divided into four main classes: Ectomyelois ceratoniae, Colletotrichum spp., Sunburn, and Healthy fruits, as shown in Fig. 1. The images were manually labeled and validated by a botanical expert, as shown in Table 2.

**Table 2:** Summary of disease classes and symptoms.

Class	Disease Cause	Scientific Name	Symptom Description	Type
**Ectomyelois ceratoniae**	Fruit borer insect	*Ectomyelois ceratoniae*	Internal decay, entry holes, frass	Insect
**Colletotrichum spp.**	Fungal infection	*Colletotrichum* spp.	Lesions, dark concentric rings, anthracnose symptoms	Fungal
**Sunburn**	Environmental stress	Abiotic, Sunburn Necrosis	Black spots, dry skin on the sun-exposed side	Environmental
**Healthy**	No visible disease	–	Smooth skin, no discoloration or lesions	Control

### 3.1 Ectomyelois ceratoniae (insect – fruit borer)

Ectomyelois ceratoniae, commonly referred to as the carob moth, is a significant pest affecting pomegranate fruits in the Middle East and surrounding regions. The larva penetrates the fruit skin and feeds internally, leading to internal decay, entry holes, and visible frass (insect excrement) around the infection site. Infestations are particularly problematic in mature fruits and often go undetected until the damage becomes severe. This pest is a key target for integrated pest management strategies due to its economic impact [9].

### 3.2 *Colletotrichum* spp. *(fungal – anthracnose)*

Anthracnose, caused by Colletotrichum species, is among the most common and destructive fungal diseases affecting pomegranate fruits. The infection typically presents as dark brown to black sunken lesions or hardened spots, often accompanied by concentric ring patterns on the fruit surface. Under warm and humid environmental conditions, these lesions can enlarge and merge, eventually leading to fruit rot and facilitating secondary infections. The pathogen is capable of surviving on plant debris and can spread through rain splash or contaminated agricultural tools, making effective disease management particularly challenging [6].

### 3.3 Sunburn (abiotic disorder – sunburn necrosis)

Sunburn is a non-infectious physiological disorder that occurs when the skin is exposed to intense and prolonged sunlight, especially during peak summer temperatures. Affected fruits typically display sun-facing blackened, dry, or necrotic skin lesions, which may eventually lead to hardening of the epidermal tissue and surface cracking. Although the internal quality of the fruit often remains unaffected, the external appearance is compromised, reducing its marketability [10]. Sunburn incidence is influenced by factors such as fruit orientation, canopy structure, and environmental stress.

### 3.4 Healthy (no visible disease symptoms)

Fruits classified as ``Healthy'' serve as the control group in the dataset and are characterized by the absence of any visible biotic or abiotic disorders. These fruits exhibit uniform coloration, smooth skin texture, and no external signs of infection, damage, or sunburn. They are essential for training and validating machine learning models, particularly in binary (diseased vs. healthy) or multi-class classification settings [11].

## 4 Experimental Design, Materials and Methods

### 4.1 Data collection

The dataset was compiled through a systematic, field-based image acquisition process conducted in September 2023. Data collection was performed during the fruiting and harvest season to capture a broad range of disease symptoms under natural field conditions.

Images were captured directly in local orchards using a high-resolution SONY ILCE-7RM4 digital camera equipped with a 31 mm focal length lens, as represented in Table 1. The camera settings were configured for natural light photography, with no flash or artificial lighting applied, to preserve the authentic appearance of disease manifestations. The average original image resolution was 7952 × 4472 pixels at 350 dpi, providing sufficient detail to observe even subtle surface-level symptoms such as fungal lesions, insect entry points, or sunburn damage. During the preprocessing stage, all images were uniformly resized to a resolution of 512 × 512 pixels to maintain consistency throughout the dataset.

The selection of fruit specimens for imaging was based on visible external symptoms. Diseased samples were identified through visual inspection and confirmed with the assistance of a botanical expert. In total, 2178 original images were collected and categorized into four classes: Colletotrichum spp., Ectomyelois ceratoniae, Sunburn, and Healthy. Each image was labeled accordingly and stored in PNG format. Table 3 presents the counts of original and augmented images included in the dataset.

**Table 3:** Number of original and augmented images per class in the pomegranate disease dataset.

Class	No of Original Images	No of Augmented Images	Total	Dimension
Ectomyelois ceratoniae	555	7215	7770	512×512
Colletotrichum spp.	571	7423	7994	512×512
Sunburn	391	5083	5474	512×512
Healthy	661	8593	9254	512×512
Total	2178	28,314	30,492	

**Fig. 2:**
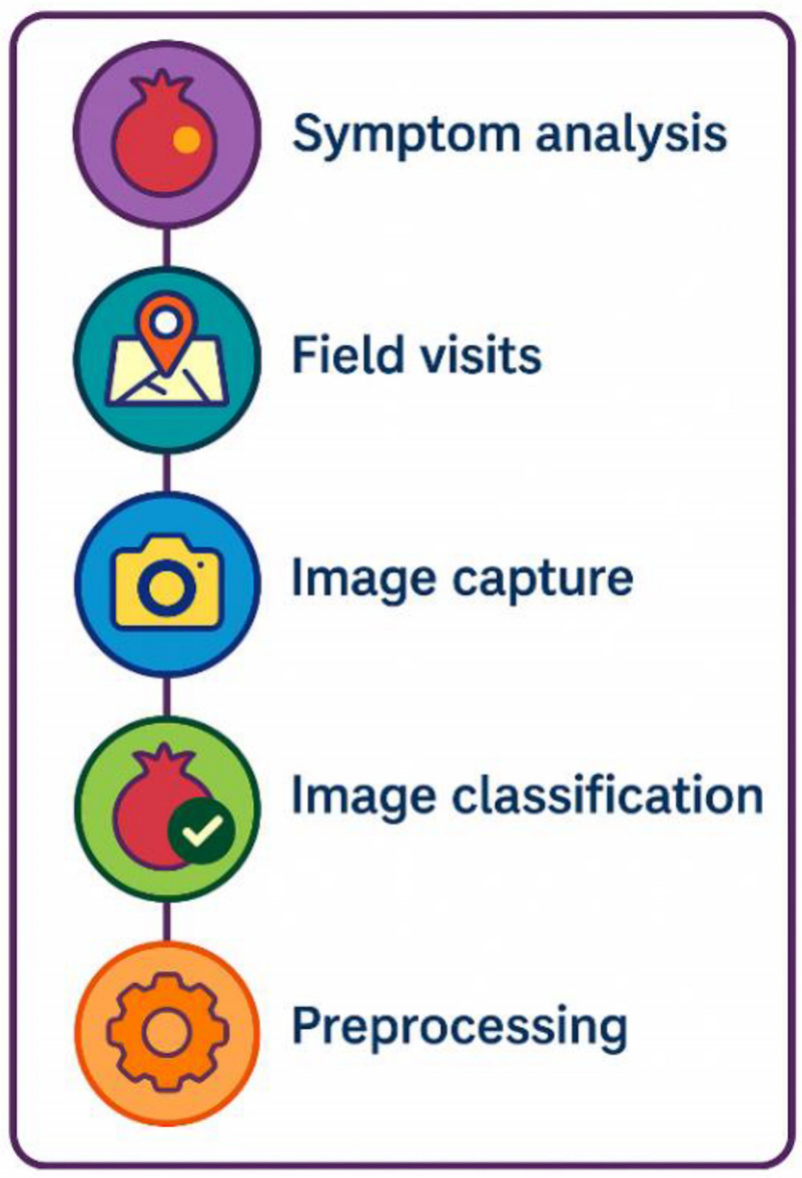
Methodological workflow for dataset preparation.

To enhance diversity and preserve the authenticity of the dataset, images were captured under a range of environmental conditions, including variations in sunlight intensity, camera angles, and background vegetation, reflecting the natural variability present in agricultural settings. A limited number of images displayed minor imperfections, such as shadows, slight motion blur, or uneven terrain; however, these were deliberately retained to represent the real-world complexity of field environments and to support the development of more robust machine learning models. The Methodological Workflow for Preparing the Pomegranate Disease Image Dataset is shown in Fig. 2.

**Fig. 3:**
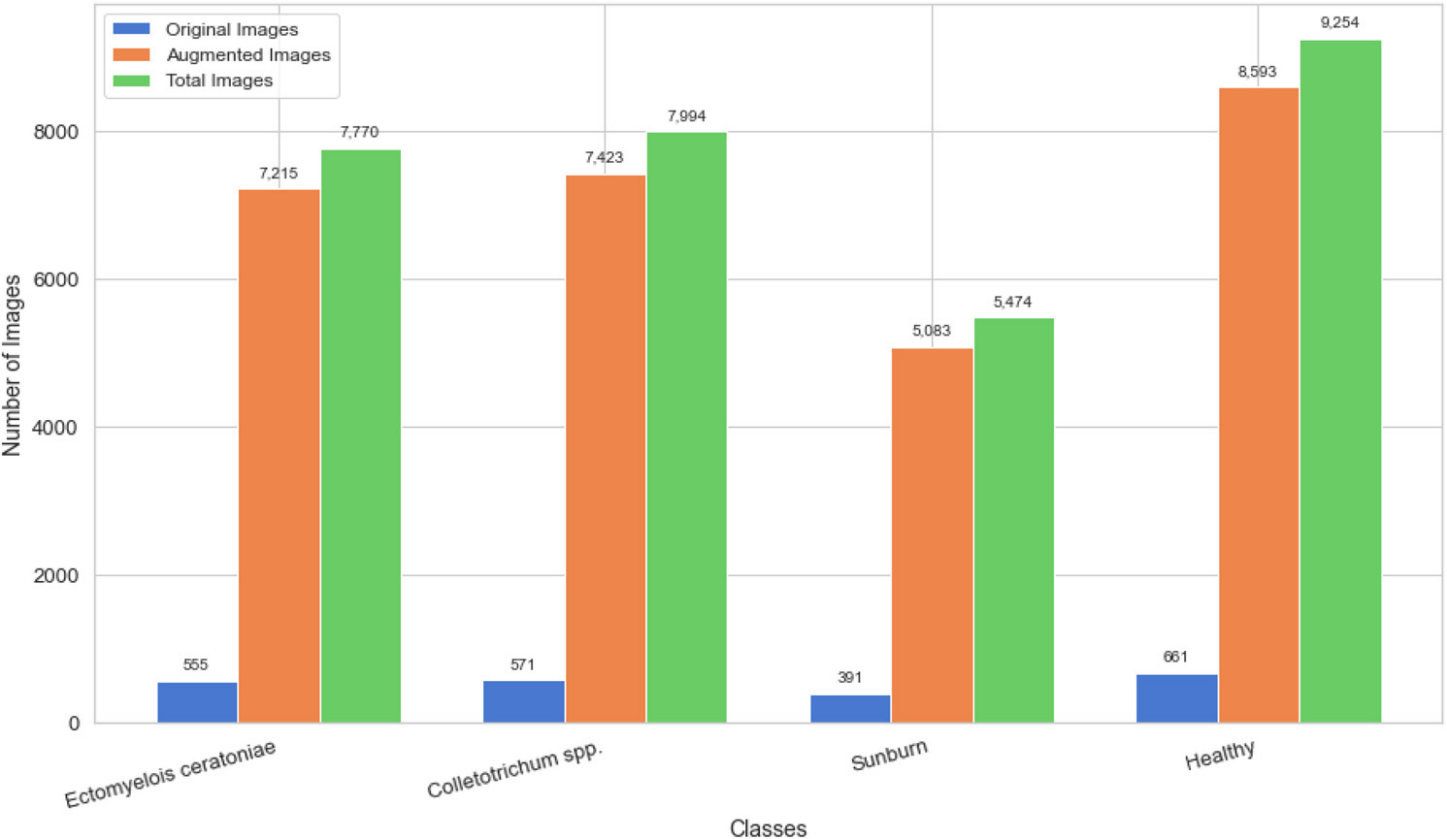
Distribution of original and augmented images across different classes.

Fig. 3 presents a comparative analysis of the number of original and augmented images for each pomegranate fruit disease class within the dataset. The ``Healthy'' class contains the highest number of images, followed by *Colletotrichum* spp. and *Ectomyelois ceratoniae*, reflecting the dataset’s emphasis on diverse disease conditions. The significant increase in image numbers after augmentation underscores the dataset’s readiness for deep learning applications that require large sample sizes.

The dataset was constructed from five separate orchards within Halabja city. Although no formal randomization protocol was employed, images were manually sampled from diverse zones within each orchard to ensure maximum representation. All images were then pooled into a unified dataset to ensure sufficient class balance and minimize cohort bias.

### 4.2 Image augmentations

**Fig. 4:**
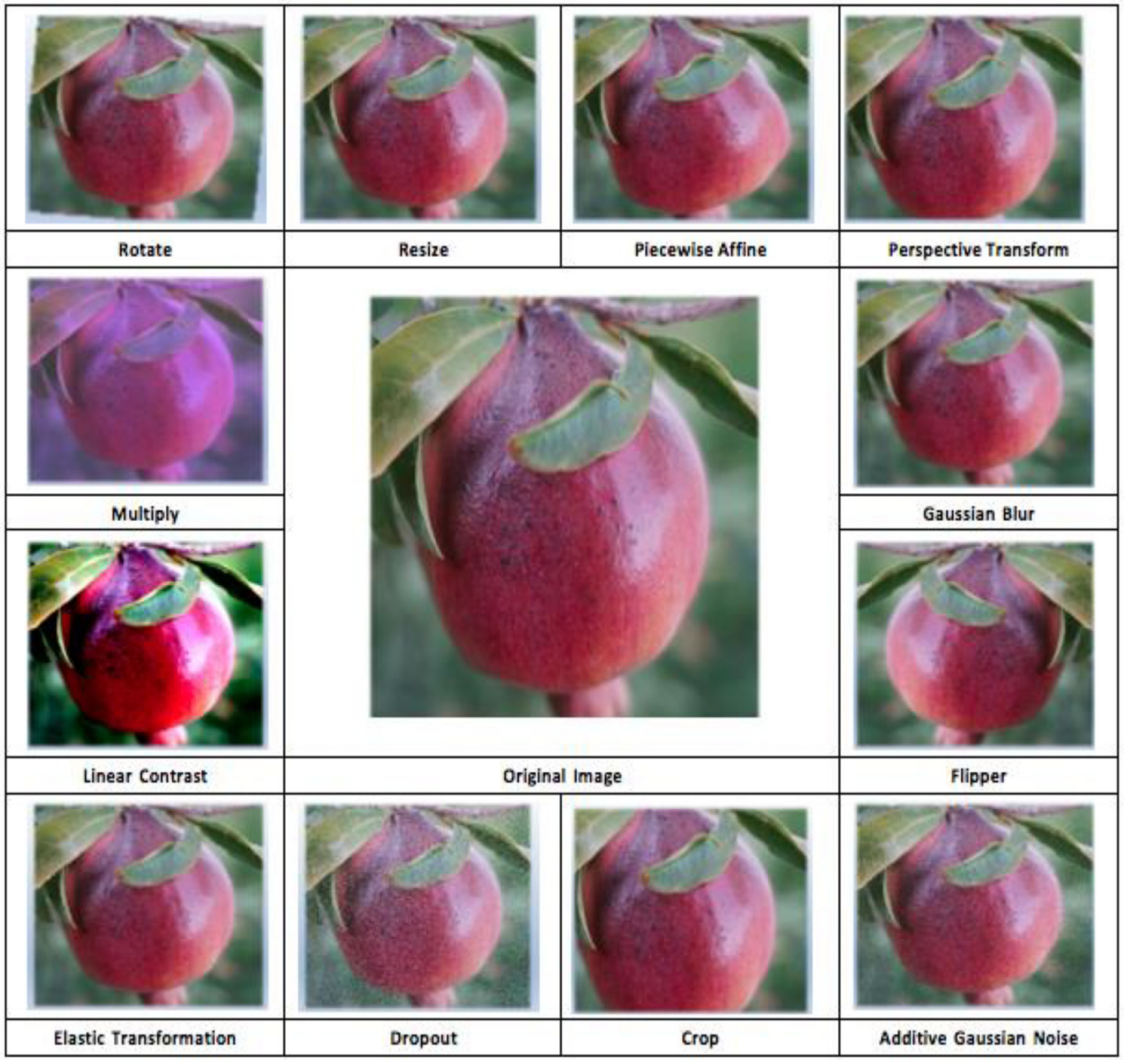
Dataset augmentation process.

To enhance the diversity and robustness of the dataset and to expand its size, a set of image augmentation techniques was applied to each original image, as illustrated in Fig. 4. This step is crucial for improving model performance and generalization in fruit image analysis, particularly for both machine learning and deep learning applications.

The selection of augmentation methods was designed to simulate various real-world conditions encountered in fruit imagery, thereby increasing the model’s ability to generalize effectively. Each image was augmented using all twelve techniques in a consistent and deterministic manner. These include Rotation, Resizing, Piecewise Affine, Perspective Transformation, Multiplication, Gaussian Blur, Linear Contrast, Flipping, Elastic Transformation, Dropout, Cropping, and Additive Gaussian Noise. This augmentation process addressed the limitations posed by data scarcity and contributed to enhancing model performance during the training, evaluation, and testing phases [12].

In this dataset, a systematic and structured image labeling strategy has been employed to ensure traceability and clarity. Each original image is assigned a unique identifier consisting of the disease class followed by a sequential number, such as ``Colletotrichum_Spp_0004''. This format allows for easy identification of the disease type and the specific image instance. For data augmentation, twelve additional images were generated from each original image using various transformation techniques commonly used in computer vision tasks. The name of the applied augmentation technique is appended to the original image label to distinguish between different versions clearly. For example, images generated from ``Colletotrichum_Spp_0004'' include ``Colletotrichum_Spp_0004_LinearContrast'', ``Colletotrichum_Spp_0004_Multiply'', ``Colletotrichum_Spp_0004_PerspectiveTransform'', and others such as ``PiecewiseAffine'', ``AdditiveGaussianNoise'', ``Crop'', ``Dropout'', ``ElasticTransformation'', ``Fliplr'', ``GaussianBlur'', ``Resize'', and ``Rotate''. This approach not only preserves the connection between original and augmented images but also facilitates efficient dataset management and reproducibility in machine learning experiments.

To further aid reproducibility, Table 4 presents the exact parameter settings used for each augmentation technique. These were selected based on best practices from the literature and were applied uniformly across all samples [13,14].

**Table 4:** Augmentation techniques and parameters applied using the imgaug library to enhance dataset diversity and model robustness.

Augmentation Technique	Function (imgaug)	Parameters	Values
Horizontal Flip	iaa.Fliplr	p (probability of applying)	1.0
Rotation	iaa.Affine	rotate	±25°
Resizing	iaa.Resize	size	(512, 512)
Piecewise Affine	iaa.PiecewiseAffine	scale	(0.01, 0.05)
Perspective Transformation	iaa.PerspectiveTransform	scale	(0.01, 0.1)
Multiplication (Brightness)	iaa.Multiply	mul	(0.8, 1.2)
Gaussian Blur	iaa.GaussianBlur	sigma	(0.0, 1.0)
Linear Contrast	iaa.LinearContrast	alpha	(0.75, 1.5)
Elastic Transformation	iaa.ElasticTransformation	alpha, sigma	alpha=50.0, sigma=5.0
Dropout	iaa.Dropout	p (dropout probability per pixel)	(0.01, 0.05)
Cropping	iaa.Crop	percent	(0, 0.1)
Additive Gaussian Noise	iaa.AdditiveGaussianNoise	scale	(0, 0.05×255)

### 4.3 Directory structure and shared files

**Fig. 5:**
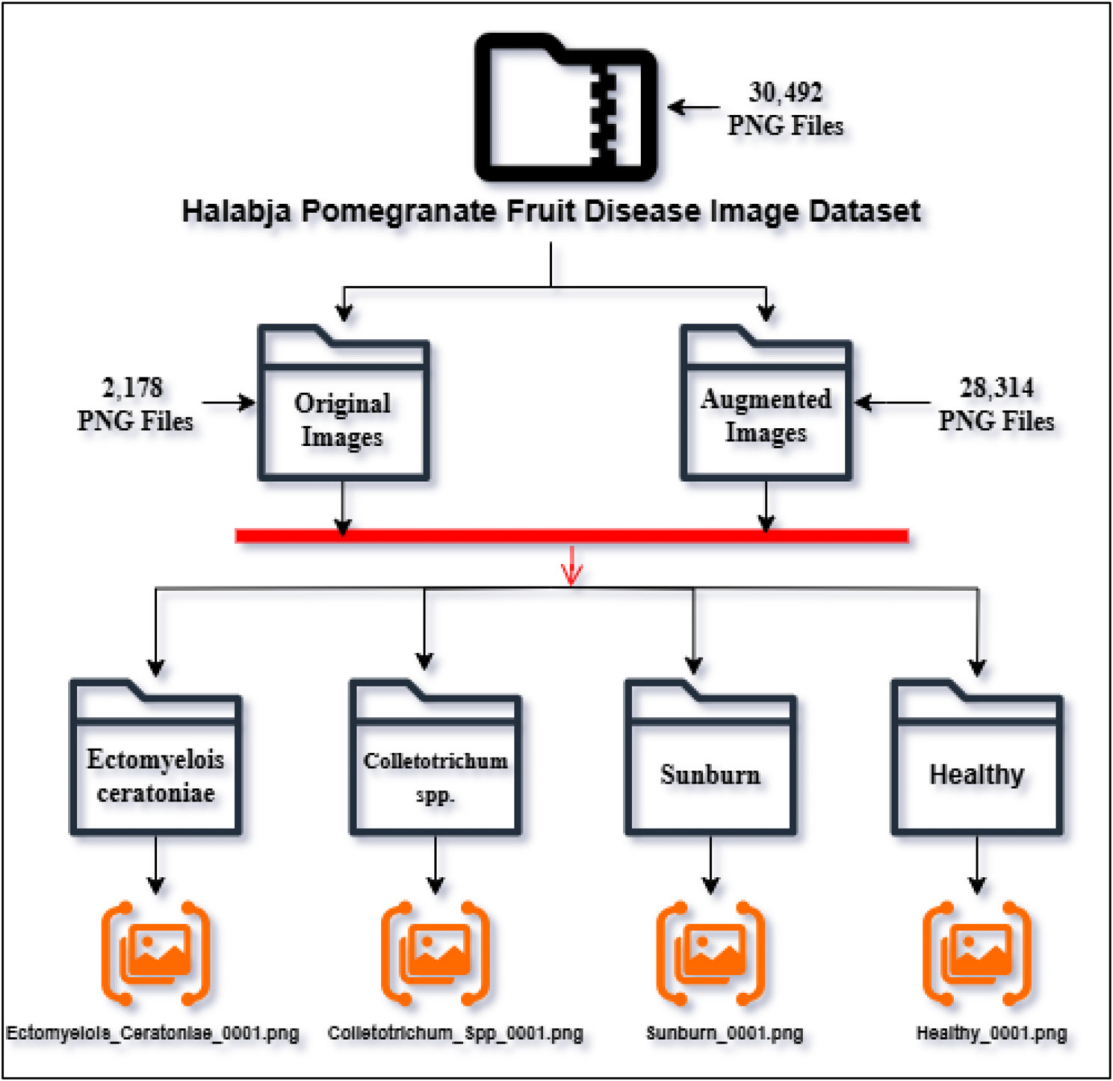
Pomegranate dataset file shared structure.

The dataset is organized into a clearly defined folder hierarchy to facilitate streamlined access and usability for machine learning and computer vision applications. The root directory, titled ``Halabja Pomegranate Fruit Disease Image Dataset,'' contains all 30,492 images of pomegranate fruits, which are systematically grouped into four subfolders, each representing a distinct classification category. Each subdirectory corresponds to a specific condition of the fruit, whether healthy or affected by a particular disease. The structure of the dataset’s file system is shown in Fig. 5.

## Limitations

While this study provides a valuable contribution to the field of plant disease identification and dataset development, several limitations should be acknowledged. Access to orchards and diseased pomegranate fruits was restricted by seasonal variability and farmer availability. The identification of diseases required expert input, which was limited and occasionally delayed the annotation process. Environmental factors such as lighting and weather, which standard camera equipment sometimes fails to capture fine details, also posed challenges. Additionally, although efforts were made to collect samples from multiple locations within Halabja, metadata such as GPS coordinates and cultivar type were not recorded, and orchard ID systems are currently not available in the Kurdistan Region of Iraq, limiting traceability. These specific imaging and sampling constraints are not expected to significantly impact model training or hinder the development of accurate and generalizable computer vision models using the dataset. Given that only a small portion of the images were affected, they were intentionally retained to preserve the realism and variability inherent in real-world agricultural conditions.

## Ethics Statement

The proposed data does not involve any human subjects, animal experiments, or data collected from social media platforms.

## CRediT Author Statement

**Bashdar Abdalrahman Mohammed:** Conceptualization, Data curation, Methodology, Visualization, Investigation, validation, Project administration, Writing, Original draft preparation. **Peshraw Ahmed Abdalla:** Data Curation, Investigation, validation, Writing, Review & Editing. **Sirwan M Aziz:** Investigation, validation, Writing. **Hiwa Omar Hassan:** Investigation, Visualization, Data-augmented Images, Writing.

## Declaration of Generative AI and AI-assisted Technologies in the Writing Process

During the preparation of this work, the authors used ChatGPT in order to improve the reliability and writing. After using this tool, the authors reviewed and edited the content as needed and take full responsibility for the content of the publication.

## Data Availability

ZENODOHalabja Pomegranate Fruit Disease Image Dataset (Original data)
